# Endoscopic Sleeve Gastroplasty: A Qualitative Narrative Review of Outcomes, Safety, and Clinical Applications

**DOI:** 10.7759/cureus.102399

**Published:** 2026-01-27

**Authors:** Mark Salib, John Salib, Frederick Tiesenga

**Affiliations:** 1 School of Medicine, St. George’s University, St. George’s, GRD; 2 Department of General Surgery, West Suburban Medical Center, Chicago, USA

**Keywords:** bariatric endoscopy, bariatric procedure selection, comparative bariatric outcomes, endoscopic bariatric therapy, endoscopic sleeve gastroplasty, endoscopic suturing, gastric volume reduction, metabolic syndrome, minimally invasive bariatric procedures, obesity management

## Abstract

Endoscopic sleeve gastroplasty (ESG) has emerged as a minimally invasive intervention for obesity, bridging the gap between conservative medical therapy and surgical bariatric procedures. ESG reduces gastric volume through endoscopic suturing, creating a tubular gastric sleeve without gastric resection and with the preservation of native anatomy. This approach offers clinically meaningful weight loss, metabolic improvement, and a favorable safety profile while minimizing procedural risk and recovery time. This qualitative narrative review provides a comprehensive evaluation of ESG, including its historical development, procedural technique, patient selection criteria, mechanisms of action, clinical outcomes, and safety considerations. The comparative efficacy of ESG relative to established bariatric surgeries, including laparoscopic sleeve gastrectomy (LSG) and open sleeve gastrectomy (OSG), is discussed, highlighting ESG’s unique balance between effectiveness and minimally invasive design. Evolving applications are also explored, including combination with pharmacologic therapy, use as a bridge to surgery for high-risk patients, and revisional strategies for weight regain after prior non-resective procedures, where preserved gastric anatomy allows safe and effective volume modification. Future directions focus on procedural refinement, optimizing patient selection, combining with adjunct therapies, and integrating into multidisciplinary obesity management. ESG represents a versatile, patient-centered tool that complements existing treatment paradigms, expands therapeutic options, and offers a practical, low-risk alternative for patients seeking minimally invasive treatment for obesity. Its growing adoption underscores its potential to play a central role in contemporary obesity management strategies.

## Introduction and background

Obesity now affects over one billion people worldwide, representing approximately one in eight individuals and underscoring its status as a major global health challenge [1]. Obesity continues to represent a significant global public health challenge, with prevalence increasing despite advances in lifestyle modification and pharmacologic therapy [1]. It is strongly associated with metabolic syndrome, cardiovascular disease, nonalcoholic fatty liver disease, obstructive sleep apnea, and increased all-cause mortality [1,2]. Although bariatric surgery remains the most effective and durable treatment for achieving sustained weight loss and metabolic improvement, it is significantly underutilized [1,3]. Barriers to surgical intervention include perceived operative risk, financial and access limitations, and patient reluctance toward permanent anatomical alteration [1-3]. Consequently, a substantial therapeutic gap persists between conservative medical management and surgical bariatric procedures [2,4].

Endoscopic bariatric therapies (EBTs) have emerged as minimally invasive interventions designed to address this gap. Among these, endoscopic sleeve gastroplasty (ESG) has garnered considerable attention as an incisionless, restrictive procedure that reduces gastric volume without requiring tissue resection or the implantation of permanent devices [2,5]. Using endoscopic suturing platforms, ESG remodels the stomach into a tubular configuration, offering a potentially reversible approach with a favorable safety profile compared to traditional bariatric surgery [1,3,4].

Since its initial introduction, ESG has evolved from an investigational technique to a widely adopted clinical intervention in specialized centers worldwide. Early studies established its feasibility and safety while demonstrating clinically meaningful short-term weight loss [2-5]. More recent investigations have expanded the evidence base to include mid- and long-term outcomes, metabolic effects, and the durability of weight loss. ESG is now increasingly considered not only as a primary therapeutic option for select patients with obesity but also as a bridge to bariatric surgery, a revisional strategy for post-surgical weight regain, and an adjunct to contemporary anti-obesity pharmacotherapy [4-6].

Despite growing utilization, heterogeneity persists in reported outcomes, technical approaches, and patient selection criteria across studies [4]. Furthermore, direct comparisons between ESG and established surgical bariatric procedures, particularly laparoscopic sleeve gastrectomy (LSG), continue to evolve as longer-term and real-world data emerge [5,6]. A contemporary synthesis of the available literature is therefore necessary to clarify the role of ESG within modern obesity treatment algorithms and to delineate its strengths, limitations, and areas requiring further investigation.

This qualitative narrative review aims to provide a comprehensive evaluation of endoscopic sleeve gastroplasty by examining its historical development, procedural and technical considerations, mechanisms of action, clinical outcomes, safety profile, and long-term durability. Additionally, this review discusses patient selection, compares ESG to surgical bariatric interventions, and highlights emerging applications and future directions in the evolving management of obesity.

History and background

Bariatric surgery remains the most effective treatment for obesity, achieving durable weight loss and significant metabolic improvements [1]. However, its invasive nature, cost, and limited accessibility have left a treatment gap between conservative medical therapy and surgical intervention [2,4]. To address this unmet need, minimally invasive endoscopic bariatric therapies (EBTs) were developed as intermediary options that can provide meaningful weight reduction with lower procedural risk [3,5].

Early EBTs included intragastric balloons and endoluminal restrictive procedures, such as transoral gastric volume reduction. These interventions demonstrated short-term efficacy and favorable safety profiles but were limited by temporary effects, device intolerance, and inconsistent long-term outcomes [2-5]. These early experiences, however, established the feasibility of endoscopic gastric remodeling and informed the development of more durable interventions.

Endoscopic sleeve gastroplasty (ESG) emerged in the early 2010s as a refinement of these approaches, utilizing full-thickness endoscopic suturing to create a sleevelike gastric configuration without resection or permanent implants [6]. Initial studies demonstrated high technical success, meaningful short-term weight loss, and a low incidence of serious adverse events, highlighting its potential as a minimally invasive alternative to surgical sleeve gastrectomy [5,7].

Over time, ESG has undergone technical refinements, including standardized suturing patterns and improved patient selection strategies, which have contributed to broader adoption across specialized centers [6]. Emerging evidence now supports its use not only as a primary therapy but also as a bridge to bariatric surgery, a revisional strategy for post-surgical weight regain, and a complement to modern pharmacologic anti-obesity therapies [8]. These developments underscore ESG’s evolving role as a durable, safe, and clinically versatile option in contemporary obesity management.

## Review

Methods

**Figure 1:**
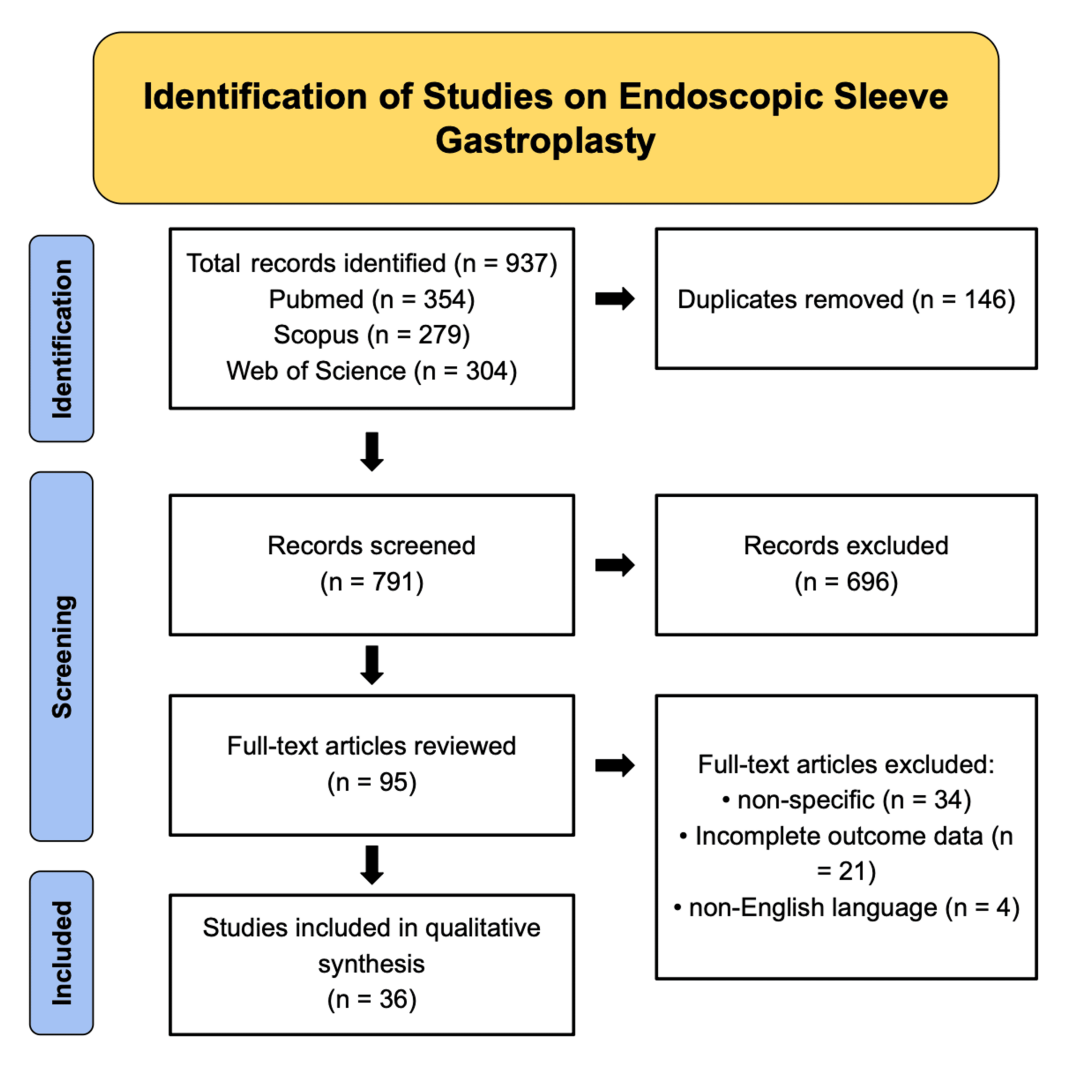
PRISMA Flow Diagram of Study Selection on Endoscopic Sleeve Gastroplasty. Flowchart illustrating the systematic identification, screening, eligibility, and inclusion process for studies evaluating endoscopic sleeve gastroplasty. A total of 937 records were identified through database searching, including PubMed (n = 354), Scopus (n = 279), and Web of Science (n = 304). After the removal of 146 duplicates, 791 records were screened by title and abstract, of which 696 were excluded. Ninety-five full-text articles were assessed for eligibility, and 59 articles were excluded due to non-specific relevance (n = 34), incomplete outcome data (n = 21), or articles that are non-English (n = 4). A total of 36 studies were included in the final qualitative synthesis. PRISMA: Preferred Reporting Items for Systematic Reviews and Meta-Analyses

This study is a qualitative narrative review designed to synthesize current evidence on the applications, outcomes, and safety profile of endoscopic sleeve gastroplasty (ESG) in the management of obesity. The review was conducted in accordance with methodological principles adapted from the Preferred Reporting Items for Systematic Reviews and Meta-Analyses (PRISMA) guidelines to ensure transparency and reproducibility (as depicted in Figure 1). A comprehensive literature search was performed across PubMed, Scopus, Embase, and Google Scholar from January 2010 to October 2025. The search strategy combined Medical Subject Headings (MeSH) and free-text keywords, including “endoscopic sleeve gastroplasty,” “ESG,” “endoscopic bariatric therapy,” “bariatric endoscopy,” “obesity,” “weight loss,” “metabolic outcomes,” “gastric remodeling,” “minimally invasive obesity treatment,” “endoscopic suturing,” and “bariatric outcomes.” Reference lists of relevant articles were manually screened to identify additional eligible studies.

The inclusion criteria comprised studies reporting outcomes of ESG in adult patients with obesity, including original clinical studies (randomized controlled trials, prospective or retrospective cohort studies, case-control studies, narrative reviews, systematic analyses, and case series), as well as secondary evidence such as systematic reviews, meta-analyses, literature reviews, and narrative reviews. Eligible studies were required to report clinical or procedural outcomes, including weight loss metrics (total body weight loss {TBWL}, excess weight loss {EWL}, and body mass index {BMI} reduction), metabolic parameters (glycemic control, blood pressure, and lipid profile), procedural safety, adverse events, or patient-reported outcomes. Studies had to be published in English in peer-reviewed journals. Exclusion criteria included abstracts, editorials, commentaries, conference proceedings, animal or preclinical studies, duplicate publications, and studies not primarily focused on ESG or endoscopic restrictive bariatric procedures. Studies assessing only pharmacologic therapy or purely surgical procedures without ESG comparison were also excluded.

Three reviewers independently screened all retrieved titles and abstracts. Full texts of potentially eligible articles were assessed for inclusion, and any disagreements were resolved by consensus discussion. Data extracted included study design, sample size, patient characteristics, procedural details, follow-up duration, clinical outcomes, and key findings. Methodological quality was assessed using design-appropriate tools, including the Cochrane Risk of Bias Tool for randomized trials, the Newcastle-Ottawa Scale for observational studies, and the Scale for the Assessment of Narrative Review Articles (SANRA) criteria for narrative reviews. Inter-reviewer agreement was quantified using Cohen’s κ coefficient. The extracted data were tabulated and summarized in Table 1.

**Table 1:** Summary of Key References on Endoscopic Sleeve Gastroplasty and Endoscopic Bariatric Therapies. Summary of study design, patient population, and reported clinical outcomes from published reviews, meta-analyses, cohort studies, and guideline statements evaluating endoscopic sleeve gastroplasty and related bariatric therapies. Outcomes reported include measures of weight loss, metabolic parameters, safety events, the durability of effect, healthcare utilization, and long-term follow-up where available [1-36].

First Author and Year	Study Design	Population	Outcomes Reported
Espinet-Coll et al. (2025) [1]	Semi-systematic review	Adults with obesity	Short-term and long-term total body weight loss; excess weight loss; changes in glycemic control, blood pressure, and lipid profile; incidence of adverse events; the durability of effect; and need for reintervention
Jerez Diaz et al. (2025) [2]	Narrative review	Patients with metabolic dysfunction-associated steatotic liver disease	Changes in body weight, liver enzyme levels, hepatic steatosis, insulin sensitivity markers, glycemic control, cardiovascular risk factors, and procedural safety
Wu et al. (2025) [3]	Narrative review	Adults with obesity	Reported ranges of total body weight loss; improvements in fasting glucose, glycated hemoglobin, blood pressure, and lipid parameters; and peri-procedural adverse events
Goddard (2025) [4]	Narrative review	Adults with obesity	Technical success rates, the magnitude of weight loss, peri-procedural complications, recovery duration, and follow-up outcomes
Zhai et al. (2025) [5]	Narrative review	Adults with obesity	Reported efficacy outcomes, weight loss durability, metabolic parameter changes, safety signals, and emerging device performance
Tripathi et al. (2025) [6]	Comparative narrative review	Adults with obesity	Total body weight loss, excess weight loss, the remission or improvement of metabolic comorbidities, complication rates, length of hospital stay, and recovery
Moutzoukis et al. (2025) [7]	Narrative review	Adults with obesity	Procedural feasibility, technical outcomes, early weight loss, safety outcomes, and emerging technology performance
Vargas et al. (2025) [8]	Systematic review	Adults with obesity	Pooled estimates of total body weight loss, excess weight loss, adverse event rates, need for revisional procedures, and follow-up duration
Pelizzo et al. (2025) [9]	Cohort study	Adolescents with obesity	Changes in body mass index percentiles, total body weight loss, safety outcomes, impact on growth, pubertal development, and nutritional status
Nigro et al. (2025) [10]	Comparative cohort study	Adults with obesity	Weight loss at 12 months; changes in glycated hemoglobin, lipid profile, and blood pressure; the remission of obesity-related comorbidities; and adverse events
Ghusn et al. (2025) [11]	Narrative review	Adults with obesity	Variability in weight loss response, metabolic outcomes, predictors of response, and outcomes with combined endoscopic and pharmacologic approaches
Mocanu et al. (2025) [12]	Retrospective database analysis	More than 500,000 patients with obesity	Thirty-day morbidity, readmission rates, emergency department utilization, length of stay, mortality, and early postoperative weight change
Hüttl and Stauch (2025) [13]	Narrative review	Adults with obesity	Long-term weight loss outcomes, metabolic improvement, complication profiles, and comparative surgical outcomes
Hanscom et al. (2025) [14]	Systematic review and meta-analysis	Patients with metabolic syndrome	Pooled changes in total body weight, glycated hemoglobin, systolic and diastolic blood pressure, lipid profile, and waist circumference
Allencherril and McCarty (2025) [15]	Narrative review	Adults with obesity	Clinical weight loss outcomes, metabolic parameter changes, safety outcomes, and durability across endoscopic therapies
Bi and Jirapinyo (2025) [16]	Narrative review	Adults with obesity	Weight loss efficacy, patient selection criteria, metabolic effects, adverse events, and follow-up outcomes
Ng and Teoh (2025) [17]	Narrative review	Adults with obesity	Reported weight loss ranges, the durability of response, metabolic outcomes, and procedural safety
Stier et al. (2025) [18]	Systematic review	Adults with obesity	Indications for therapy, the magnitude of weight loss, metabolic improvement, adverse event rates, and durability
Baratte et al. (2025) [19]	Guideline and position statement	Adults with obesity	Recommended outcome thresholds, expected weight loss ranges, safety benchmarks, and follow-up outcome reporting standards
Lahooti et al. (2025) [20]	Prospective cohort study	Adults with obesity	Five-year total body weight loss, maintenance changes in metabolic comorbidities, quality of life scores, and revision rates
Walradt and Jirapinyo (2025) [21]	Narrative review	Adults with obesity	Longitudinal outcomes including weight maintenance, metabolic disease control, and safety over time
Král and Machytka (2024) [22]	Narrative review	Adults with obesity	Evolution of clinical outcomes, weight loss durability, the refinement of technique, and safety reporting
Zhu et al. (2025) [23]	Systematic review and network meta-analysis	Adults with overweight and obesity	Comparative weight loss efficacy, adverse event rates, and durability across endoscopic and surgical modalities
Ying et al. (2025) [24]	Narrative review	Adults with obesity	Weight loss outcomes, metabolic improvements, complication rates, and procedural considerations
Mrad et al. (2025) [25]	Network meta-analysis of randomized trials	Adults with obesity	Comparative magnitude of weight loss, the durability of effect, adverse events, and treatment ranking
Dayyeh et al. (2024) [26]	Evidence-based position statement	Adults with obesity	Expected weight loss outcomes, metabolic benefits, safety benchmarks, and follow-up outcome measures
Diab et al. (2024) [27]	Meta-analysis	Adults with obesity	Weight loss efficacy, adverse event rates, durability, and comparative outcomes
Gala et al. (2024) [28]	Narrative review	Adults with obesity	Device-related technical success, procedural outcomes, safety, and early weight loss
Lahooti et al. (2024) [29]	Narrative review	Adults with obesity	Role of endoscopic therapies in long-term weight management, adjunctive strategies, and outcome sustainability
Walradt and Thompson (2024) [30]	Narrative review	Adults with obesity	Procedural learning curve, weight loss outcomes, safety profile, and follow-up results
Abuawwad et al. (2024) [31]	Commentary review	Adults with obesity	Indications, clinical outcomes, safety considerations, and expected weight loss
Pessorrusso et al. (2024) [32]	Narrative review	Adults with obesity	Weight loss magnitude, metabolic improvements, safety outcomes, and durability
Aderinto et al. (2023) [33]	Narrative review	Adults with obesity	Surgical weight loss outcomes, metabolic effects, complication rates, and long-term results
Abdulla et al. (2023) [34]	Narrative review	Adults with obesity	Efficacy and safety outcomes of endoscopic weight loss therapies
Winder and Rodriguez (2023) [35]	Narrative review	Adults with obesity	Early clinical outcomes, weight loss feasibility, and safety
Docimo et al. (2023) [36]	Systematic review	Adults with obesity	Weight loss durability, metabolic outcomes, adverse events, and need for revision

Given the heterogeneity in study design, patient populations, and outcome measures, a qualitative narrative synthesis was performed. Findings were organized thematically into procedural technique, patient selection, clinical efficacy, safety, comparative outcomes with surgical procedures, and emerging applications of ESG.

Procedure overview/technique

Endoscopic sleeve gastroplasty (ESG) is a minimally invasive, incisionless procedure performed using a flexible endoscope and a full-thickness endoscopic suturing system, most commonly the OverStitch platform (Apollo Endosurgery, Austin, TX) [6]. The procedure is typically performed under general anesthesia with endotracheal intubation to ensure airway protection and patient comfort [2,5]. ESG is conducted via the oral route, avoiding abdominal incisions and permanent gastric alteration.

The fundamental goal of ESG is to reduce gastric volume and alter gastric motility by creating a tubular, sleevelike configuration [6]. After initial diagnostic endoscopy, a series of full-thickness sutures is placed along the greater curvature of the stomach, typically from the antrum to the gastroesophageal junction, using a running or interrupted pattern [6,8]. Standard suture configurations include the “U-shaped” or “triangular” plication pattern, which approximates the anterior and posterior gastric walls to create durable gastric folds [6]. The number of sutures is variable, often ranging from six to 12, depending on the gastric anatomy and the operator’s preference [6-8].

Procedural success is assessed by the visual confirmation of the sleeve formation and functional luminal reduction, generally achieving a 70%-80% reduction in gastric volume [6-8]. Carbon dioxide insufflation is preferred to minimize post-procedural discomfort and reduce the risk of complications such as pneumoperitoneum [5]. Procedural duration typically ranges from 60 to 90 minutes in experienced centers, with the learning curve showing improvements in efficiency and technical success over the first 20-30 cases [6,9].

ESG is often combined with peri-procedural measures to optimize outcomes, including prophylactic antibiotics, careful hemostasis during suture placement, and post-procedural dietary counseling [8,9]. Unlike surgical sleeve gastrectomy, ESG preserves the gastric fundus and does not involve stapling or tissue resection, allowing for potential reversibility or revision if needed [9]. Modifications in suture patterning and the number of plications have been explored to enhance durability, improve weight loss outcomes, and reduce adverse events [8,9].

**Figure 2:**
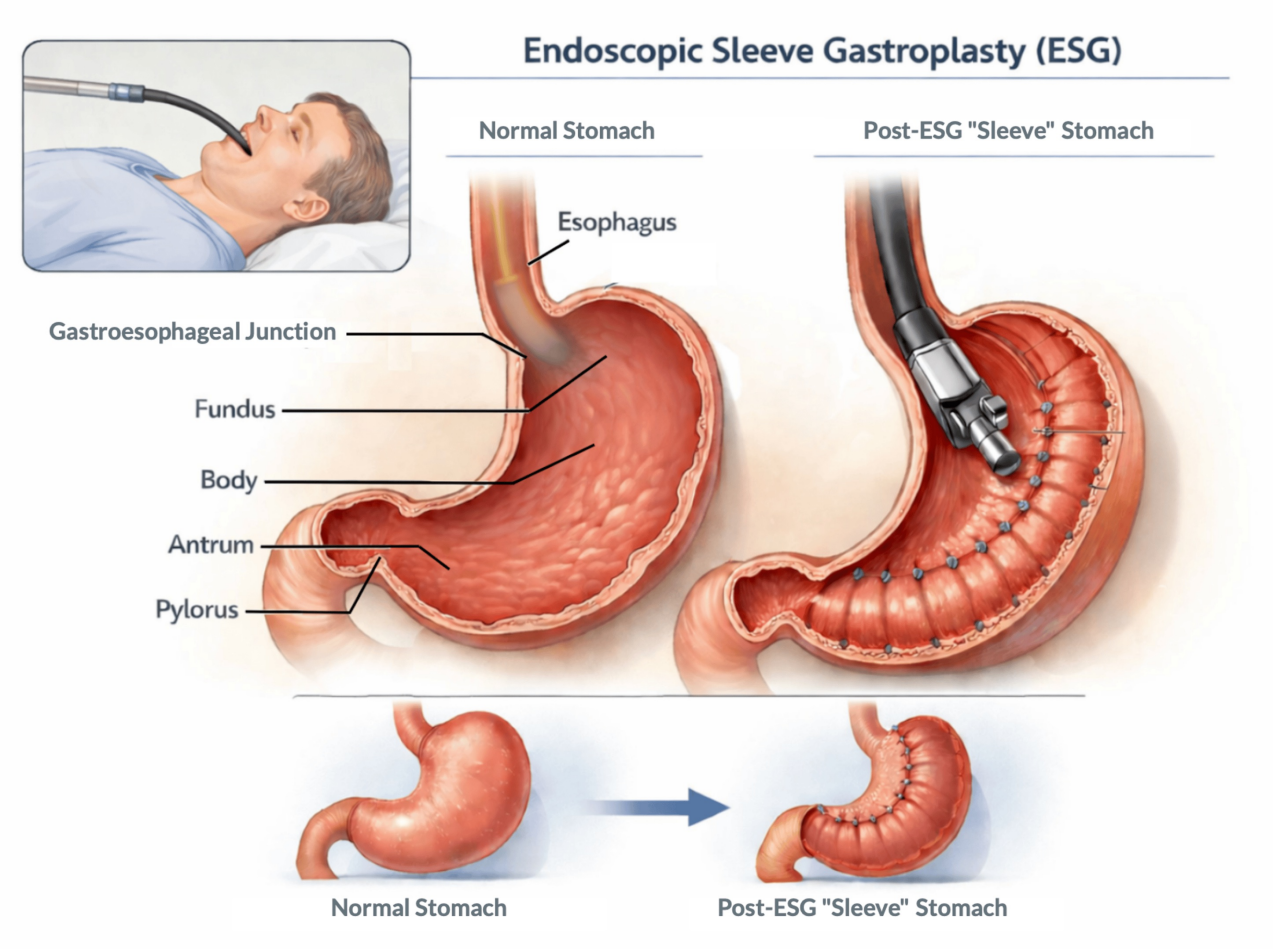
Endoscopic Sleeve Gastroplasty (ESG) Procedure. Illustration of ESG in a supine patient under general anesthesia. The endoscope is inserted orally into the esophagus, and full-thickness sutures are placed along the greater curvature of the stomach. Sequential plications create a tubular sleeve along the lesser curvature, reducing gastric volume. Key structures include the stomach, gastroesophageal junction (GEJ), pylorus, and tabular sutures [2-10]. Images designed and created by the authors.

Overall, ESG (as depicted in Figure 2) represents a technically reproducible, minimally invasive procedure that leverages endoscopic suturing to achieve meaningful gastric restriction and functional remodeling without permanent anatomical alteration, offering an attractive alternative to surgical sleeve gastrectomy in appropriately selected patients.

Indications and patient selection

Endoscopic sleeve gastroplasty (ESG) is indicated for adults with obesity seeking a minimally invasive alternative to bariatric surgery or those not eligible for surgery [7,8]. Current evidence supports the use of ESG in patients with a body mass index (BMI) of 30-40 kg/m², who have documented failure of lifestyle modification or pharmacotherapy and achieved 15%-20% total body weight loss at 12 months in most series [7-9]. Patients with a BMI of >40 kg/m² may also be considered, often as a bridge to surgery or in combination with medical therapy to reduce perioperative risk [9,10].

Candidate selection should incorporate comorbidity assessment, including type 2 diabetes, hypertension, dyslipidemia, and nonalcoholic fatty liver disease [4,11]. ESG has been shown to improve metabolic parameters, including reductions in glycated hemoglobin (HbA1c) and blood pressure, which supports its use in patients seeking both weight reduction and comorbidity management [9,12]. Ideal candidates are motivated, able to adhere to post-procedural lifestyle changes, and free of contraindications to endoscopy or general anesthesia [12]. Contraindications include severe gastroesophageal reflux disease, large hiatal hernia, prior gastric surgery altering anatomy, coagulopathy, or active peptic ulcer disease. Psychiatric assessment may also be considered to ensure realistic expectations [10-13].

Emerging indications for ESG include bridge therapy for high-risk surgical candidates, revisional therapy for post-bariatric weight regain, and combination with pharmacologic therapies, such as glucagon-like peptide-1 (GLP-1) receptor agonists, to optimize weight loss and metabolic outcomes [10-14]. The consideration of gastric anatomy, prior surgical history, and behavioral readiness is essential in tailoring patient selection. Shared decision-making remains critical, balancing procedural efficacy, safety, and patient preferences to maximize both short- and long-term outcomes.

Safety profile

Endoscopic sleeve gastroplasty (ESG) is generally well-tolerated, with a favorable safety profile compared to surgical bariatric procedures [10,13]. Across multiple prospective and multicenter studies, serious adverse events are reported in approximately 1%-2% of cases, while minor adverse events such as transient pain, nausea, or vomiting occur in 15%-20% of patients [14]. Most minor complications are self-limited and resolve within a few days with conservative management.

The most common serious complications include gastrointestinal bleeding, perigastric fluid collections, and, rarely, gastric perforation [15]. Perigastric collections are typically managed conservatively with antibiotics or endoscopic drainage, and perforation, though infrequent, may necessitate surgical intervention [14-16]. Pulmonary complications are uncommon but can occur, primarily related to anesthesia in high-risk patients [16].

Compared to laparoscopic sleeve gastrectomy, ESG avoids stapling, gastric resection, and permanent anatomical alteration, contributing to lower rates of severe complications, shorter procedural times, and faster recovery [15,17]. Additionally, ESG does not appear to increase the risk of nutritional deficiencies commonly associated with malabsorptive bariatric procedures, although adherence to post-procedural dietary guidance remains critical [17].

Operator experience and procedural volume are important determinants of safety. Centers with established ESG programs report reduced complication rates and improved technical efficiency, highlighting the role of standardized training and experience in minimizing adverse events [18,19]. Careful patient selection, including the evaluation of comorbidities, prior abdominal surgery, and gastric anatomy, further reduces procedural risk.

Overall, ESG demonstrates a high safety profile with low rates of serious adverse events, supporting its role as a minimally invasive option for weight reduction in appropriately selected patients. Vigilant peri-procedural monitoring, adherence to technical protocols, and structured post-procedural follow-up remain crucial to optimizing outcomes and ensuring safety.

Clinical outcomes

Endoscopic sleeve gastroplasty (ESG) has consistently demonstrated clinically meaningful and durable outcomes across short-, medium-, and long-term follow-up, establishing it as a central tool in contemporary obesity management. In the short term (6-12 months), ESG achieves total body weight loss (TBWL) of 15%-20% and excess weight loss (EWL) of 50%-60%, with reductions in BMI averaging 5-7 kg/m² [19-21]. Patients also experience significant metabolic improvements, including reductions in HbA1c (0.8%-1.2%), fasting glucose, triglycerides, low-density lipoprotein cholesterol, systolic blood pressure, and inflammatory markers such as C-reactive protein, reflecting the procedure’s impact on both weight and cardiometabolic risk [19-22]. Procedural success rates are high (>95%), and the incidence of serious adverse events is low, supporting ESG as a safe and effective intervention in the early post-procedural period [19,20,23].

Medium-term outcomes (12-24 months) demonstrate the maintenance of 13%-18% TBWL and sustained metabolic benefits [23]. Longitudinal studies report that 80%-85% of patients retain ≥10% TBWL at 24 months, with most comorbidities, including type 2 diabetes and hypertension, remaining improved [23,24]. Patient-reported outcomes also indicate increased satiety, improved mobility, and enhanced quality of life, highlighting ESG’s broader functional and psychosocial impact [23,24]. A modest weight regain may occur in a subset of patients, underscoring the importance of adhering to structured dietary, behavioral, and follow-up programs.

Long-term outcomes, extending up to five years in emerging data, suggest durable weight reduction in 50%-60% of patients, with continued improvement in metabolic markers and low rates of revisional intervention [25]. Weight regain, when present, is generally modest and may be mitigated through adjunct pharmacologic therapy, such as GLP-1 receptor agonists, or repeat endoscopic plication [24,26]. Predictors of long-term success include a lower baseline BMI, greater adherence to lifestyle modifications, an optimal suture pattern and number, an early response to ESG, and the combination with pharmacotherapy [24-26].

When compared to intensive lifestyle modification or pharmacologic therapy alone, ESG consistently achieves superior weight loss and metabolic improvement [25]. Relative to laparoscopic sleeve gastrectomy, ESG produces slightly lower absolute weight loss but offers advantages including a minimally invasive approach, the preservation of gastric anatomy, lower complication rates, shorter recovery times, and the potential for reversibility [27]. Collectively, these data (as depicted in Table 2) establish ESG as a practical, durable, and versatile intervention, providing meaningful clinical, metabolic, and quality-of-life benefits across short-, medium-, and long-term horizons.

**Table 2:** Clinical Outcomes of Endoscopic Sleeve Gastroplasty Across Short-, Medium-, and Long-Term Follow-Up. Overview of ESG outcomes across short-, medium-, and long-term follow-up, highlighting meaningful weight loss, sustained metabolic benefits, and a favorable safety profile. ESG offers a minimally invasive, reversible alternative to laparoscopic sleeve gastrectomy, combining efficacy with lower complication risk and faster recovery [19-27]. ESG, endoscopic sleeve gastroplasty; TBWL, total body weight loss; EWL, excess weight loss; LSG, laparoscopic sleeve gastrectomy; BP, blood pressure; LDL, low-density lipoprotein; HbA1c, glycated hemoglobin; CRP, C-reactive protein; BMI, body mass index; GLP-1, glucagon-like peptide-1

Follow-Up Period	Weight Outcomes	Metabolic and Clinical Outcomes	Procedural Success and Safety	Key Clinical Insights
Short term (6-12 months)	TBWL, 15%-20%; EWL, 50%-60%; BMI, ↓ 5-7 kg/m²	HbA1c, ↓ 0.8%-1.2%; fasting glucose, ↓ 10-15 mg/dL; triglycerides, ↓ 20-35 mg/dL; LDL, ↓ 10-15 mg/dL; systolic BP, ↓ 5-10 mmHg; CRP, ↓ 1-2 mg/L	Procedural success, >95%; serious adverse events, 1%-2%	ESG produces rapid weight reduction and cardiometabolic improvements with high procedural success and minimal serious complications, supporting its role as a safe, minimally invasive intervention [19-23]
Medium term (12-24 months)	TBWL, 13%-18%; ≥10% TBWL retained in 80%-85%	HbA1c, ↓ 0.7%-1.0%; triglycerides, ↓ 15-25 mg/dL; LDL, ↓ 8-12 mg/dL; BP improvements maintained	Serious adverse events: 1%-3%	Weight loss and metabolic benefits are sustained; patients report improved satiety, mobility, and quality of life. Modest weight regain may occur, emphasizing structured dietary and behavioral follow-up [23,24]
Long term (up to five years)	Durable TBWL in 50%-60%	HbA1c, ↓ 0.5%-0.8%; LDL and triglycerides remain improved	Revisional interventions: 5%-10%	Long-term durability is supported in half of the patients, with continued metabolic benefit. Adjunct pharmacotherapy (e.g., GLP-1 receptor agonists) or repeat ESG can mitigate weight regain. Predictors of success include lower baseline BMI, adherence to lifestyle changes, optimal suture pattern, and early response [24-26]
Comparative perspective	Slightly lower TBWL than LSG (25%-30%)	Superior to intensive lifestyle or pharmacologic therapy alone	Minimally invasive, preserves gastric anatomy, lower complication rates, faster recovery, and reversible	ESG provides a clinically meaningful alternative to surgery, particularly for patients who are high-risk, prefer minimally invasive therapy, or require gastric preservation. Benefits include lower perioperative risk and procedural flexibility [25-27]

Comparison to laparoscopic and open sleeve gastrectomy (OSG)

Endoscopic sleeve gastroplasty (ESG) offers a minimally invasive, incisionless alternative to laparoscopic sleeve gastrectomy (LSG) and open sleeve gastrectomy (OSG), providing clinically meaningful weight loss while preserving gastric anatomy and minimizing procedural risks. In terms of efficacy, ESG achieves total body weight loss (TBWL) of 15%-20% at 12 months and excess weight loss (EWL) of 50%-60%, with sustained TBWL of 10%-15% at 24-36 months [28-30]. LSG achieves higher weight reduction, with 25%-30% TBWL and 65%-75% EWL at 12 months, while OSG yields slightly higher values (TBWL, 30%-35%; EWL, 70%-80%) [28,29]. Despite lower absolute weight loss, ESG demonstrates durable outcomes, particularly in patients with moderate obesity (BMI: 30-40 kg/m²) or those seeking a less invasive approach.

Metabolic outcomes follow a similar hierarchy. ESG consistently improves glycemic control (HbA1c reduction: 0.8%-1.2%), blood pressure, and lipid profile, although LSG generally produces greater long-term reductions in HbA1c (≈1%-1.5%) and other cardiometabolic risk markers due to more substantial weight loss [30,31]. OSG outcomes are comparable to LSG but are associated with higher perioperative morbidity [28,30]. While Roux-en-Y gastric bypass (RYGB) and biliopancreatic diversion with duodenal switch (BPD/DS) produce the most pronounced metabolic benefits through both restrictive and malabsorptive mechanisms, ESG offers clinically meaningful improvements without the nutritional risks associated with malabsorptive surgery [31].

Safety and perioperative recovery are key differentiators. ESG has a serious adverse event rate of 1%-2%, with minor complications such as transient nausea, epigastric pain, or mild post-procedure bleeding in 15%-20% of patients [30-32]. LSG carries a 5%-10% risk of serious complications, including staple-line leaks, hemorrhage, or nutritional deficiencies, whereas OSG exhibits a higher perioperative risk (10%-15%) due to the invasiveness of the open approach [30,32,33]. ESG is typically performed outpatient or with <24 hours of hospitalization, allowing return to daily activities within 1-3 days, compared with 1-2 days of hospitalization and 2-4 weeks of recovery after LSG and 4-7 days of hospitalization with 4-6 weeks of recovery after OSG [30-35].

Another key advantage of ESG is its reversibility and procedural flexibility. Gastric anatomy is preserved, allowing for repeat endoscopic plication in cases of weight regain and combination with adjunct pharmacologic therapy (e.g., GLP-1 receptor agonists) [33,34]. Surgical sleeves are permanent, and revisions require higher technical complexity with increased risk [34,36]. ESG can also serve as a bridge to surgery for high-risk patients, enabling preoperative weight loss to reduce operative risk [36].

In summary, as depicted in Table 3, ESG occupies a middle ground in the bariatric spectrum. It achieves substantial, durable weight loss and metabolic improvement with a low complication profile, faster recovery, and procedural flexibility. While LSG and OSG offer greater absolute weight reduction and long-term metabolic benefit, ESG provides a safer, minimally invasive alternative, particularly for patients with moderate obesity, higher surgical risk, or a preference for non-permanent interventions. Its versatility positions ESG as a complementary and increasingly relevant option in contemporary obesity management.

**Table 3:** Comparative Overview of Endoscopic Sleeve Gastroplasty, Laparoscopic Sleeve Gastrectomy, and Open Sleeve Gastrectomy. Overview of clinical, metabolic, safety, and procedural parameters for endoscopic sleeve gastroplasty (ESG), laparoscopic sleeve gastrectomy (LSG), and open sleeve gastrectomy (OSG). Parameters include total body weight loss (TBWL), excess weight loss (EWL), metabolic outcomes (HbA1c, blood pressure {BP}, and lipid profile), serious adverse event rates, recovery time, reversibility/flexibility, and typical patient selection [28-36]. HbA1c: glycated hemoglobin

Features	Endoscopic Sleeve Gastroplasty (ESG)	Laparoscopic Sleeve Gastrectomy (LSG)	Open Sleeve Gastrectomy (OSG)
Weight loss (12 months)	TBWL, 15%-20%; EWL, 50%-60% [28-30]	TBWL, 25%-30%; EWL, 65%-75% [28-30]	TBWL, 30%-35%; EWL, 70%-80% [28-30]
Weight loss (24-36 months)	TBWL, 10%-15%; durable in moderate obesity [28-30]	Greater absolute reduction; sustained [28-30]	Comparable to LSG but higher perioperative morbidity [28-30]
Metabolic outcomes	HbA1c, ↓ 0.8%-1.2%; BP and lipids improved [30,31]	HbA1c, ↓ 1%-1.5%; stronger long-term reductions [30,31]	Similar to LSG but higher perioperative risk [30,31]
Serious adverse events	1%-2%; minor events 15%-20% [30-32]	5%-10%; includes leaks, hemorrhage, and nutritional deficiencies [30-32]	10%-15%; higher due to open approach [30-33]
Recovery/hospitalization	Outpatient or <24 hours; return to activity in 1-3 days [30-35]	1-2 days of hospitalization; 2-4 weeks of recovery [30-35]	4-7 days of hospitalization; 4-6 weeks of recovery [30-35]
Reversibility/flexibility	Preserves anatomy; repeat plication or adjunct pharmacotherapy possible; bridge to surgery [33-36]	Permanent; revisions more complex [33-36]	Permanent; higher technical complexity for revisions [33-36]
Patient selection/advantages	Moderate obesity, higher surgical risk, and preference for minimally invasive or reversible procedure [28-36]	Higher BMI, desire for greater weight loss, and tolerates surgery [28-36]	Severe obesity or complex cases where an open approach is indicated [28-36]

Future directions

Endoscopic sleeve gastroplasty (ESG) has established itself as a minimally invasive option for managing obesity; however, several areas warrant further investigation to define its long-term role and optimize clinical outcomes. Future studies should focus on the durability of weight loss and metabolic improvements, particularly in comparison to established surgical procedures, to better understand ESG’s position within the spectrum of obesity therapies.

Procedural innovation remains a key frontier. Advances in suture patterns, anchoring devices, and integration with robotic or enhanced endoscopic platforms have the potential to improve procedural efficiency, minimize complications, and enhance efficacy. Concurrently, adjunctive therapies, including pharmacologic agents such as GLP-1 receptor agonists, may be combined with ESG to maximize weight loss and metabolic benefits, thereby expanding its applicability to a broader patient population.

Patient selection and personalization are critical for optimizing outcomes. Future research should aim to identify clinical, behavioral, and biological predictors of response to ESG, enabling tailored therapy and reducing the need for subsequent interventions. ESG may also be evaluated as a bridge therapy for high-risk patients prior to bariatric surgery or as a revisional strategy for post-surgical weight regain, with standardized protocols guiding its safe and effective use.

Finally, integrating ESG into healthcare systems and cost-effectiveness analyses is a crucial priority. Understanding how ESG fits within multidisciplinary obesity management, including its impact on recovery, procedural risk, and combination with pharmacologic therapy, will inform clinical decision-making and facilitate broader adoption.

In summary, the future of ESG lies in procedural refinement, combination therapies, improved patient selection, and strategic integration into clinical pathways. Continued research in these areas will clarify their long-term efficacy, safety, and role within comprehensive obesity management.

## Conclusions

Endoscopic sleeve gastroplasty (ESG) represents a safe, minimally invasive, and versatile intervention for obesity, occupying a unique position between conservative medical therapy and surgical bariatric procedures. This qualitative narrative review highlights ESG’s ability to produce meaningful weight loss and metabolic improvements while minimizing procedural risk, preserving gastric anatomy, and enabling faster recovery. Comparative analyses suggest that while surgical approaches, such as laparoscopic and open sleeve gastrectomy, achieve greater absolute weight loss, ESG offers a favorable balance of efficacy, safety, and procedural flexibility, making it particularly suitable for patients with moderate obesity, a higher surgical risk, or a preference for non-permanent interventions. Emerging applications, including combination with pharmacologic therapies, use as a bridge to surgery, and revisional strategies for post-surgical weight regain, underscore ESG’s expanding clinical relevance. Future research, focusing on procedural refinement, long-term outcomes, optimized patient selection, and integration into multidisciplinary care pathways, will further define its role in contemporary obesity management. In summary, ESG is a clinically effective, patient-centered tool that complements existing treatment paradigms, expands therapeutic options, and has the potential to play a central role in the evolving landscape of obesity care. Its adoption represents a meaningful advancement in minimally invasive obesity treatment strategies.
